# Non-invasive sleep EEG measurement in hand raised wolves

**DOI:** 10.1038/s41598-022-13643-x

**Published:** 2022-06-13

**Authors:** Vivien Reicher, Anna Bálint, Dóra Újváry, Márta Gácsi

**Affiliations:** 1 Department of Ethology, Doctoral School of Biology, Institute of Biology, Eötvös Loránd University, Budapest, Hungary; 2 MTA-ELTE Comparative Ethology Research Group, Budapest, Hungary; 3 Department of Ethology, Institute of Biology, Eötvös Loránd University, Budapest, Hungary

**Keywords:** Neuroscience, Cognitive neuroscience, Zoology, Animal physiology

## Abstract

Sleep research greatly benefits from comparative studies to understand the underlying physiological and environmental factors affecting the different features of sleep, also informing us about the possible evolutionary changes shaping them. Recently, the domestic dog became an exceedingly valuable model species in sleep studies, as the use of non-invasive polysomnography methodologies enables direct comparison with human sleep data. In this study, we applied the same polysomnography protocol to record the sleep of dog’s closest wild relative, the wolf. We measured the sleep of seven captive (six young and one senior), extensively socialized wolves using a fully non-invasive sleep EEG methodology, originally developed for family dogs. We provide the first descriptive analysis of the sleep macrostructure and NREM spectral power density of wolves using a completely non-invasive methodology. For (non-statistical) comparison, we included the same sleep data of similarly aged dogs. Although our sample size was inadequate to perform statistical analyses, we suggest that it may form the basis of an international, multi-site collection of similar samples using our methodology, allowing for generalizable, unbiased conclusions. As we managed to register both macrostructural and spectral sleep data, our procedure appears to be suitable for collecting valid data in other species too, increasing the comparability of non-invasive sleep studies.

## Introduction

Although not always easy to determine, sleep or sleep-like states have been described in many different species from invertebrates to fish, birds and mammals^1,2^. Studies on different species have shown that while there are important similarities between some features of sleep between species, sleep behaviour typically shows huge variations between different taxa in terms of architecture, duration and even its physiological and neurological features^1^.

We know that in humans, sleep affects the majority of our physiological (e.g. immunity, hormonal regulation, metabolism^3,4^ as well as cognitive functions (e.g. learning, memory consolidation^5^). Thus, it is no surprise that any disorder in the sleeping process can have widespread effects on our physical and mental health^6^. One way to broaden our knowledge in the science of sleep is to include different animal models in our investigations, allowing us to qualitatively and quantitatively compare the sleep of different species^7^. Since the applicability of different measurement techniques and set-ups can vary significantly between species, the use of similar methodologies in different species may prove imperative from a comparative perspective^8^.

In humans, sleep studies have mostly been conducted using non-invasive methods^3^, invasive techniques have only been used in some cases of specific medical importance (e.g. epilepsy diagnostics^4,5^; or brain-computer interface for paralyzed patients^6^). In most animal models, however, awake and/or sleep brain activations have traditionally been measured by invasive techniques (e.g. rat^7^, cat^8^, dog^9^). Although invasive methods such as surgically implanted and subcutaneous needle electrodes provide higher brain signal quality, they are associated with several considerable disadvantages. These experiments are not conducive to current ethical standards of animal experimentation and the experimental set-ups and designs are often difficult to relate to real-life settings^10–12^. Prompted in part by these rising issues, non-invasive methods to assess sleep data of model animals have also emerged in the past years. For example, electric field sensors successfully quantified wake, REM and NREM sleep in mice, although without the detection of brain signals, only macrostructural sleep variables were observable^13^. In rhesus macaques, an EEG cap method was applied to measure the awake brain functioning of the animals^11^. Although the procedure is claimed to be non-invasive, the stabilization of the head during the experiment required the surgical implantation of a head post on the animals’ dorsal cranium, restraining them during the measurements^11^. So far, fully non-invasive EEG measurements were published in a few species^14,15^, including the dog^16^. Experiments measured the awake brain activity of laboratory beagles during different visual tasks non-invasively, however, this required an 18-month-long training of the dogs’ prior to the tests (e.g.^17,18^). In another line of research, untrained family dogs were measured non-invasively in a number of different sleep EEG (e.g.^19–22^) and awake ERP^23,24^ experiments.

The increasing interest in canine sleep research stems from its advantages to study the sleep of a domesticated species adapted to the human environment^25^. As evolutionary adaptations to environmental circumstances—such as sleeping in a protected environment—might have shaped humans’ sleep (e.g. increased deepness of human sleep^26^; brain monitoring function during sleep in an unfamiliar environment^27^), similar changes might be observable in the sleep of other species adapted to the human environment. For example, findings indicated first-night-effect-like adaptation processes in dogs’ sleep^21^, similarly to humans^28^. To gain a better understanding of the effects of domestication on sleep phenotypes and physiology, comparing a species to its wild counterpart—in this case the wolf^29,30^—offers a unique opportunity. Dog-wolf comparative studies have already been conducted in several areas of research including behavioural^31,32^ and genetic studies^33,34^, however, the neural processes of wolves remain a largely unexplored field.

So far, most studies investigated the activity patterns of free-ranging wolves, using methods such as radiotelemetry^35^ and GPS collars^36^. Other studies compared the resting behaviour of captive wolves and dogs (raised and socialized equally), measuring physiological parameters such as heart rate (HR) and heart rate variability (HRV)^37,38^. While Kortekaas et al.^37^ assumed that wild species are more alert and sensitive towards their environment than their domesticated relatives based on previous reports^39,40^, they found that wolves (separated from their pack) were less alert (e.g. had lower HRs) in both inactive wakefulness and resting conditions, compared to dogs. However, another study found contrasting results, with dogs' HR being lower in the “alone” resting condition compared to wolves^38^. It should be noted that these discrepancies may primarily stem from the different experimental settings across studies and the small sample sizes used in the experiments (six wolves and six/seven dogs were included in both studies).

The daily activity of free-living wolf packs is highly variable as some of the packs move and hunt at night^41,42^ while others are more active during the day^43,44^. Possible reasons for this variability include human disturbance^41,42^, variation in the amount and distribution of food over time^45^, reproductive activity^45^, and fluctuating weather conditions^35^. In contrast, captive wolves have been characterized by daytime activity and nighttime sleep (e.g.^46^), which is similar to family dogs’ daily activity^47^. In our own experience in the present study, the wolves’ caregiver (living on site), described the wolves’ circadian rhythm as diurnal, generally adapting to the human-controlled environment (e.g. conforming to the feeding regime and training sessions during the day while settling down during the night).

In this study, we present the first step to provide non-invasive sleep EEG data in untrained, captive individuals of a wild species. We non-invasively measured the sleep EEG of wolves, using the same methodology as has extensively been applied in family dogs. Specifically, we hypothesized that we can successfully mount the surface electrodes according to the canine EEG protocol and we can measure at least one full sleep cycle (cc. 30 min) including a REM phase and at least 10 min of artefact-free NREM traces, allowing for the spectral analysis of the sleep EEG. For comparison, sleep data of similarly aged dogs are also included.

## Method

### Subjects

The total sample of grey wolves (Canis lupus) was N = 7, all of them living at the Horkai Animal Training Center at Gödöllő (https://horkai.com/hu/). Our subjects included six young wolves between the age of 4–5 months (Mage = 3.9, SD = 0.5) from the same litter and one adult wolf at the age of 13 years (for details see Supplementary Table 1). All wolves were individually hand-reared from the age of 4–14 days up to the age of 3 months (see e.g.^48^). After this age, they were accommodated at the Horkai Animal Training Center, living together in packs. Throughout the time with their caretakers, wolves were intensively socialized including frequent encounters with stranger humans and dogs as well as being familiarized with novel locations and situations on a regular basis.

At the Training Centre, fourteen well socialized wolves lived at the time of the experiment. Only highly socialized, healthy wolves were included in the measurements who were also familiar (i.e. familiar from the date of birth, with weekly meetings) with at least one of the experimenters (N = 7). These criteria caused the gap in the age range, as none of the experimenters were familiar with the adult wolves (between 1 and 13 years of age).

The total sample of family dogs was N = 20 including 10 puppies and 10 senior dogs from the same age-range as the corresponding sample of young and senior wolves. Since selective breeding of dogs might have affected the developmental trajectories of breeds differently^49^, possibly affecting their sleep EEG as well, we selected a sample of various different breeds and mongrels in our study. They were selected from a database of previously published dog polysomnography experiments^50,51^. The 10 puppies were between the age of 3–5 months (Mage = 3.9, SD = 0.9; all purebreds from 9 different breeds; 5 females; all intact). The 10 seniors were at the age of 13 years (5 purebreds from 3 different breeds; 6 females; all neutered). For details see Supplementary Table S1.

### Ethical statement

This research was approved by the Hungarian “Animal Experiments Scientific and Ethical Committee” (PE/EA/853–2/2016; PE/EA/865-5/2021) and was conducted in accordance with Hungarian regulations on animal experimentation and Guidelines for use of animals in research, as outlined by the Association for the Study Animal Behaviour (ASAB). Please note that In Hungary, we have a two-level ethical process. First, our animal experimentation methodology was reviewed and approved by the Animal Welfare Committee of Eötvös Loránd University (ELTE). Second, the Ethics Committee of our university (ELTE) forwarded our ethical request to the higher governmental office (National Food Chain Safety Office of Hungary) which also reviewed and approved it (reference number PE/EA/853–2/2016; PE/EA/865-5/2021). The owners participated voluntarily without monetary compensation and provided their signed an informed consent.

### Experimental location and procedure

The sleep recordings in both dogs and wolves were conducted in an unfamiliar room, after a relatively active day (e.g., an excursion requiring physical activity), starting between 12 and 8 p.m.

Dogs were measured in the canine sleep laboratories of the Family Dog Project, suitable for conducting basic sleep recordings. Equipped with only a mattress and a reading lamp for the comfort of both the owner and the dog, they provide a calm, dark and quiet environment for the dog to settle and fall asleep, while the experimenter controlled the data acquisition from outside of the laboratory. The dogs had access to water ad libitum throughout the measurements. Signa Spray Electrode Solution was used to separate the dogs’ and wolves’ hair where the Gold-coated Ag/AgCl electrodes were attached to the skin using EC2 Grass Electrode Cream (Grass Technologies, USA). For the detailed procedure of the electrode placement on young dogs, see^51^. While the electrode placement on senior dogs is detailed in this study^21^. The first sleep attempt was successful in all dogs.

The Senior wolf was measured in our canine sleep laboratory, while the young wolves were measured in a 2 × 3 m, unfamiliar room at the Horkai Animal Training Centre. The room provided a quiet environment for the sleep recordings, although it could not be completely darkened. Two people were present during the measurements, the caretaker of the given wolf and the experimenter (E), who was familiar with all of the wolves. During the measurements, first, E and the caretaker entered the room with the wolf and let the animal explore the room. After the wolf settled and/or started to fall asleep (cc. 30–60 min later), E sat next to the wolf to attach the electrodes. If the wolf became aroused, E stopped the electrode placement. The caretaker and E calmed the wolf by praising and petting it, while waiting for the wolf to settle again. If the attempt to secure the electrodes was unsuccessful for more than 90 min (e.g. over-excited wolf, noise from outside) we aborted the measurement and let the wolf back to its pack. While the first measurement attempt was successful in the case of 5 wolves, only the second attempts were successful in the remaining 2 animals.

Additionally, we collected data from a second sleep occasion from 3 wolves: Wolf 4 (at the age of 6 months), Wolf 6 (at the age of 8 months) and Senior wolf (one month after the first measurement). The second attempt was successful on the first occasion for Wolf 4 and Senior wolf, while it was only successful on the third occasion for Wolf 6. Unfortunately, due to the mating season starting in winter, all measurements had to be aborted after December, firmly limiting the amount of data we could gather. The animals became unsettled and agitated, rendering sleep measurements impossible. Later follow-up measurements were unfortunately precluded due to construction works beginning at the facility the following spring and the ensuing Covid-19 pandemic.

Behavioural signs of stress were also closely controlled throughout the measurements. Wolves were only handled by familiar people, gently caressing and petting them to sleep after some physical exercise (i.e. long walk in the forest). None of the wolves have shown signs of common stress symptoms such as becoming agitated, attempting to escape or showing aggression (see^52,53^).

**Figure 1:**
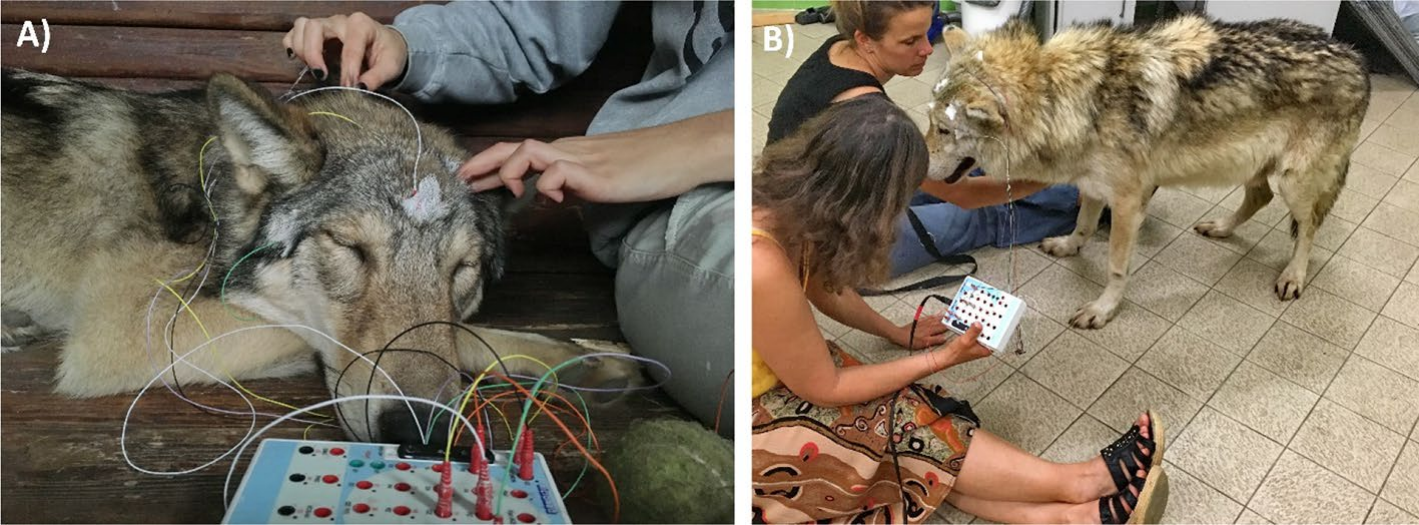
Photo of (**A**) a sleeping young wolf during electrode placement and (**B**) the Senior wolf in the lab at the Department of Ethology after electrode placement, waiting for him to lay down.

**Figure 2:**
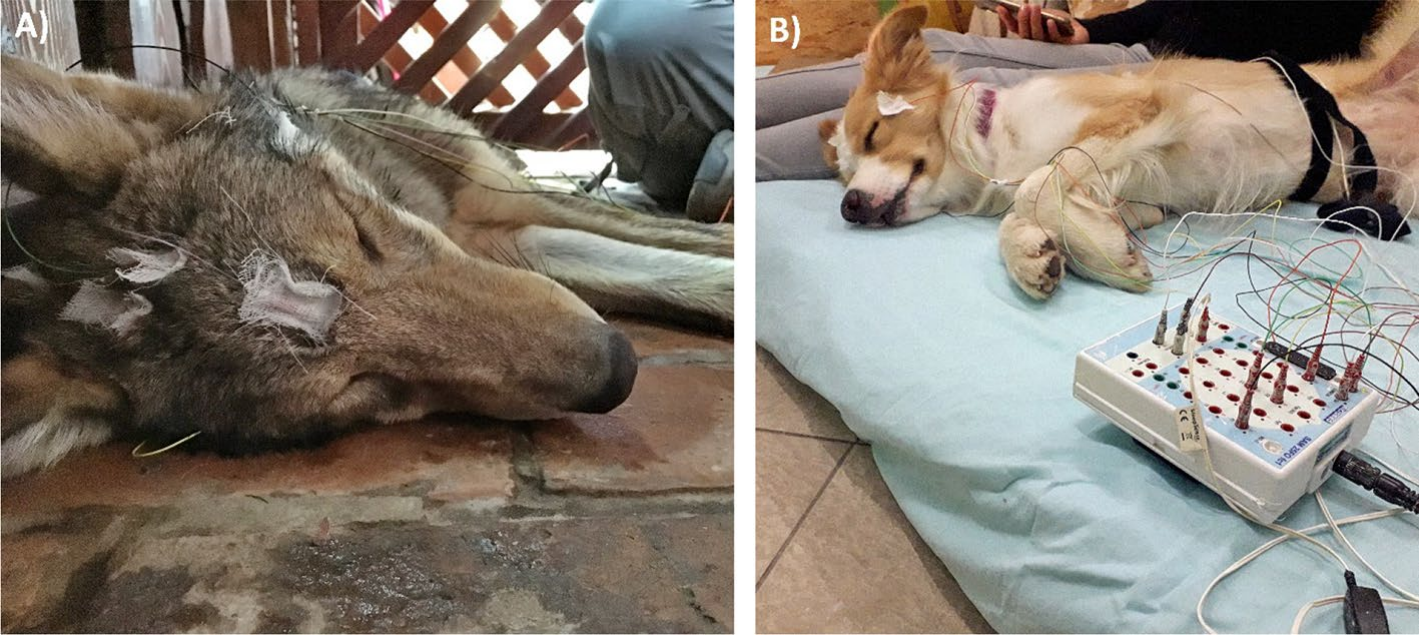
Photo of a (**A**) sleeping young wolf and (**B**) dog with electrodes (originally published in^51^).

The maximum duration of sleep recordings (i.e. the total duration of time the electrodes were attached to the animals’ head) was set to be max. 180 min, although it varied among subjects (see “Results”). Figure 1 shows wolves during electrode placement, while Fig. 2 shows a sleeping wolf and a dog with electrodes.

### Electroencephalographic (EEG) method

A detailed description of the most recent polysomnographic method and EEG electrode placement can be found in our previous study^21^ and/or see Supplementary document (see section “EEG recording”) and Supplementary Fig. S1. Recordings were obtained with two technical arrangements (as one EEG equipment was mobile, while the other was not). For details, see Supplementary document (section “EEG recording”).

### Sleep analysis

Sleep recordings were visually scored in 20-s epochs in accordance with standard criteria^54^ adapted for dogs^55^ using Fercio’s EEG Plus (© Ferenc Gombos 2009–2022). This method is suitable for the reliable identification of the following stages: wake, drowsiness, NREM (i.e. non-rapid eye movement) and REM (i.e. rapid eye movement) with a good inter-rater agreement between coders^56^, resulting in a hypnogram for each recording (see Supplementary Fig. S2 for hypnograms and Fig. S3 for the EEG traces of different sleep stages of Young wolf 1). The following sleep macrostructure variables were exported from the program: record duration, sleep duration (drowsiness + NREM + REM), drowsiness and NREM latency, duration of drowsiness, NREM and REM sleep; sleep efficiency (the percentage of time spent asleep: drowsiness + NREM + REM during the sleep measurement), the proportion of time spent in drowsiness, NREM and REM sleep. Additionally, we analyzed the number of awakenings from NREM and REM sleep.

Some animals were so active (i.e. playing) upon starting the measurements that the electrodes could only be attached once they were almost asleep or dozing off. Since the recordings started from the point when all electrodes were securely attached, the sleep latency variable of some individuals was 0. Wake after sleep onset was also dependent on subject compliance and was thus not exported. Some young animals became so active after short awakenings that the recordings had to be stopped, while others continued lying and relaxing next to their caretaker, often falling back asleep so that the recording could be continued.

Relative power spectra were calculated only for NREM sleep and only for the Fz channel, because the NREM stage provided the highest amount of artefact-free traces and the Fz channel was uniformly recorded in all dogs and wolves. Although both the dogs’ and wolves’ sleep included drowsiness and REM sleep, due to the high amount of muscle and eye movement artefacts, we refrained from the spectral power analyses of these sleep stages. Artefact rejection was carried out manually on 4 s epochs. Average power spectral densities (1 Hz to 30 Hz) were calculated by a Fast Fourier Transformation (FFT) algorithm, applied to the 50% overlapping, Hanning-tapered 4 s windows of the EEG signal of the Fz-G2 derivations. Dogs show significant individual-level variation in the morphological features of their head musculature, skull shape and thickness^57^ that might have an influence on the EEG data. To prevent a measurement error arising from these differences, absolute power was normalized by computing the relative power spectra of the delta (1–4 Hz), theta (4–8 Hz), alpha (8–12 Hz), sigma (12–16 Hz) and beta (16–30 Hz) bands of NREM sleep.

### Data analysis

Due to the small sample size, statistical analyses were not performed. We have performed power calculations (R^58^; package pwr; power calculations for two samples, different sizes). Based on the sample sizes used in the study (since *n* of each group should be larger than 1, the data of young animals were used) and assuming large effect sizes (d = 0.7, estimated from the mean effect sizes of the young animals’ data), the calculated power was low (power = 0.24).

In our study only descriptive data are presented. Tables 1, 2 show the summarized sleep data of each wolf and dog, as well as the mean and standard error of the different sleep variables in different age groups. In the case of senior dogs, we included the data of the first 59 min of sleep recordings in order to make it more comparable to the 59-min-long sleep recording of the Senior wolf.

**Table 1:** Sleep macrostructure variables of individual wolves and dogs.

Subject	Age	Record	Sleep	Drowsi. latency	Drowsi	NREM latency	NREM	REM	Awaken
Wolf 1	3.2 m	64.0	60.0	1.0	2.3	3.0	37.0	20.7	3
Wolf 2	3.7 m	62.3	55.7	0	4.0	0	36.7	15.0	6
Wolf 3	3.7 m	59.3	51.3	0	5.3	0	41.0	5.0	8
Wolf 4 a	3.7 m	77.7	66.0	1.0	4.7	2.0	42.3	19.0	4
Wolf 4 b	5.9 m	67.3	60.7	0	9.7	0	45.7	5.3	12
Wolf 5	4.4 m	92.7	83.0	0	8.3	0	47.3	27.3	11
Wolf 6 a	4.7 m	56.7	36.3	3.7	5.7	5.0	25.7	5.0	5
Wolf 6 b	7.8 m	40.3	30.6	0	3.7	0	23.3	3.6	5
Mean ± SD		68.8 ± 13.8	58.7 ± 15.6	0.7 ± 1.21	5.1 ± 2.0	1.3 ± 1.8	38.3 ± 7.3	15.3 ± 8.9	5.9 ± 2.8
Dog 1	2.5 m	92.7	83.3	1.0	11.3	2.0	58.3	13.7	14
Dog 2	2.9 m	142.0	112.7	2.7	26.3	5.7	65.0	21.3	16
Dog 3	3.3 m	139.0	115.7	7.7	7.0	8.3	84.0	24.7	12
Dog 4	3.6 m	80.7	59.7	15.3	7.0	17.7	44.0	8.7	5
Dog 5	3.8 m	180.0	110.3	9.0	25.0	9.7	71.0	14.3	11
Dog 6	3.9 m	180.0	141.0	1.3	28.3	3.0	83.0	29.7	18
Dog 7	4.6 m	32.0	27.0	1.3	1.7	8.7	18.3	7.0	3
Dog 8	4.6 m	84.0	33.7	0	6.7	1.3	27.0	0.0	2
Dog 9	5.3 m	57.7	43.7	6.3	3.3	8.0	36.0	4.3	4
Dog 10	5.3 m	123.0	100.7	4.3	9.7	11.0	69.7	21.3	9
Mean ± SD		113.2 ± 46.6	83.5 ± 38.3	4.9 ± 4.5	12.6 ± 10.1	7.5 ± 4.6	56.8 ± 21.3	14.2 ± 10.0	9.4 ± 5.7
Sen. Wolf a	13 y	58.7	33.3	15.7	9.3	20.3	8.0	15.7	2
Sen. Wolf b	13 y	67.3	49.7	10.0	14.7	15.3	30.3	4.7	4
Sen. Dog 1	13 y	59.0	21.0	3.0	13.7	24.7	7.3	0	2
Sen. Dog 2	13 y	59.0	20.7	24.7	12.3	27.3	8.4	0	18
Sen. Dog 3	13 y	59.0	36.0	1.3	12.3	7.0	22.3	1.3	13
Sen. Dog 4	13 y	59.0	30.3	12.3	21.0	15.7	8.7	0.6	9
Sen. Dog 5	13 y	59.0	46.7	2.7	33.3	8.7	13.4	0	18
Sen. Dog 6	13 y	59.0	53.0	3.0	11.3	7.3	38.67	3.0	8
Sen. Dog 7	13 y	59.0	42.3	5.7	14.7	22.3	27.6	0	6
Sen. Dog 8	13 y	59.0	44.0	9.7	18.3	14.7	24.0	1.7	12
Sen. Dog 9	13 y	59.0	24.3	7.7	22.0	44.0	2.3	0	4
Sen. Dog 10	13 y	59.0	11.7	39.3	2.7	42.3	9	0	5
Mean ± SD			33.0 ± 12.8	10.9 ± 11.5	16.2 ± 7.7	21.4 ± 12.8	16.2 ± 10.9	0.7 ± 1.0	10.0 ± 5.7

All sleep variables are given in minutes (min). Ages are shown in months (m) and years (y). Sen. indicates Senior, Awaken. indicates the number of awakenings, while the ‘a’ and ‘b’ in the subject’s name indicate the first and second sleep recordings.

**Table 2:** Relative sleep macrostructure and spectral variables of individual wolves and dogs.

Subject	Age	Sleep eff	Drowsi	NREM	REM	Delta	Theta	Alpha	Sigma	Beta
Wolf 1	3.2 m	93.8	3.9	61.7	34.4	91.9	6.1	1.0	0.3	0.7
Wolf 2	3.7 m	89.3	7.2	65.9	26.9	89.7	7.3	2.0	0.6	0.4
Wolf 3	3.7 m	86.5	10.4	79.9	9.7	88.6	7.6	2.3	0.8	0.8
Wolf 4 a	3.7 m	85.0	7.1	64.1	28.8	86.7	9.5	2.3	0.9	0.6
Wolf 4 b	5.9 m	90.1	15.9	75.3	8.8	85.1	8.3	3.1	1.5	1.9
Wolf 5	4.4 m	89.6	10.0	57.0	32.9	88.1	8.5	2.1	0.7	0.6
Wolf 6 a	4.7 m	64.1	15.6	70.6	13.8	88.4	8.1	2.1	0.7	0.8
Wolf 6 b	7.8 m	70.8	11.9	76.1	12.0	87.3	8.5	2.1	0.9	1.1
Mean ± SD		84.7 ± 10.5	9.0 ± 4.0	66.5 ± 7.9	24.4 ± 10.3	88.9 ± 1.8	7.9 ± 1.2	1.9 ± 0.5	0.6 ± 0.2	0.7 ± 0.1
Dog 1	2.5 m	89.9	13,6	70.0	16.4	83.9	11.5	2.9	0.8	0.9
Dog 2	2.9 m	79.3	23,4	57.7	18.9	92.6	4.8	1.6	0.6	0.5
Dog 3	3.3 m	83.2	6.1	72.6	21.3	89.7	7.8	1.6	0.6	0.4
Dog 4	3.6 m	67.3	11.7	73.7	14.5	92.7	5.7	1.2	0.3	0.2
Dog 5	3.8 m	61.2	22.7	64.4	13.0	89.4	7.3	1.8	0.8	0.6
Dog 6	3.9 m	78.2	20.1	58.8	21.0	90.3	6.3	2.0	0.7	0.7
Dog 7	4.6 m	33.5	6.2	67.9	25.9	90.4	6.8	1.7	0.7	0.5
Dog 8	4.6 m	40.1	19.8	80.2	0	92.5	5.5	1.4	0.4	0.2
Dog 9	5.3 m	75.7	7.6	82.4	9.9	89.2	6.5	2.2	0.8	1.3
Dog 10	5.3 m	81.8	9.6	69.2	21.2	91.4	6.2	1.6	0.5	0.4
Mean ± SD		72.2 ± 14.3	13.8 ± 7.2	71.5 ± 9.5	15.6 ± 8.0	90.2 ± 2.6	6.8 ± 1.9	1.8 ± 0.5	0.6 ± 0.2	0.6 ± 0.3
Sen. Wolf a	13 y	56.8	28.0	24.0	48.0	58.4	19.9	7.4	4.2	10.2
Sen. Wolf b	13 y	73.8	29.5	61.1	9.4	76.4	14.7	4.5	1.8	2.5
Sen. Dog 1	13 y	35.6	65.1	34.9	0	86.8	6.7	1.9	0.8	3.8
Sen. Dog 2	13 y	34.5	60.7	39.3	0	70.1	11.5	5.8	3.2	9.4
Sen. Dog 3	13 y	61.0	34.3	62.0	3.7	78.2	12.9	4.4	1.9	2.6
Sen. Dog 4	13 y	51.4	69.2	28.6	2.2	67.3	16.0	5.2	3.6	7.9
Sen. Dog 5	13 y	79.1	71.4	28.6	0	75.7	12.1	4.8	2.5	4.9
Sen. Dog 6	13 y	89.8	21.4	73.0	5.6	79.9	3.6	1.7	1.3	2.4
Sen. Dog 7	13 y	71.8	34.6	65.4	0	83.0	10.3	3.2	1.3	2.1
Sen. Dog 8	13 y	74.6	41.7	54.5	3.8	72.3	15.7	5.6	2.3	4.2
Sen. Dog 9	13 y	41.2	90.4	9.6	0	61.3	19.0	7.8	5.2	6.7
Sen. Dog 10	13 y	19.8	22.9	77.1	0	78.2	12.9	4.4	1.9	2.6
Mean ± SD		55.9 ± 21.7	51.2 ± 22.1	47.3 ± 21.2	1.5 ± 2.0	75.3 ± 7.3	12.1 ± 4.3	4.5 ± 1.8	2.4 ± 1.2	4.7 ± 2.4

All sleep variables are given in percentage (%). Ages are indicated in months (m) and years (y). Sen. indicates Senior, while ‘a’ and ‘b’ in the subject’s name indicate the first and second sleep recordings.

## Results

We successfully conducted non-invasive sleep measurements in untrained wolves. We attached all six electrodes, similarly to the sleep measurement protocol of family dogs. We managed to register all sleep stages (drowsiness, NREM and REM). We have also successfully analyzed the power spectral density of NREM stages, using at least 10 min long, artefact-free traces. The inter-rater reliability comparison showed substantial agreement (Cohen’s κ = 0.61) between two observers based on the whole recordings of three young wolfves and a senior wolf. The length of sleep recordings were between 57 and 93 min in wolves, between 57 and 180 min in young dogs and 180 min long in senior dogs.

The excessive variability in recording length was due to differences in subject compliance. For example, young dogs were more likely to fall asleep early on during the measurement (some of the subjects fell asleep during the attachment of the electrodes). However, in case of brief awakenings during sleep, some dogs could settle again and fall back asleep, while others became so active that the recording had to be stopped, regardless of the prior time spent asleep.

### Sleep macrostructure

To better illustrate trends in the data, the figures show the first hour (± 10 min, to avoid depicting interrupted sleep phases) of sleep EEG data after the animals fell asleep, i.e. from the first drowsiness or NREM epoch.

**Figure 3:**
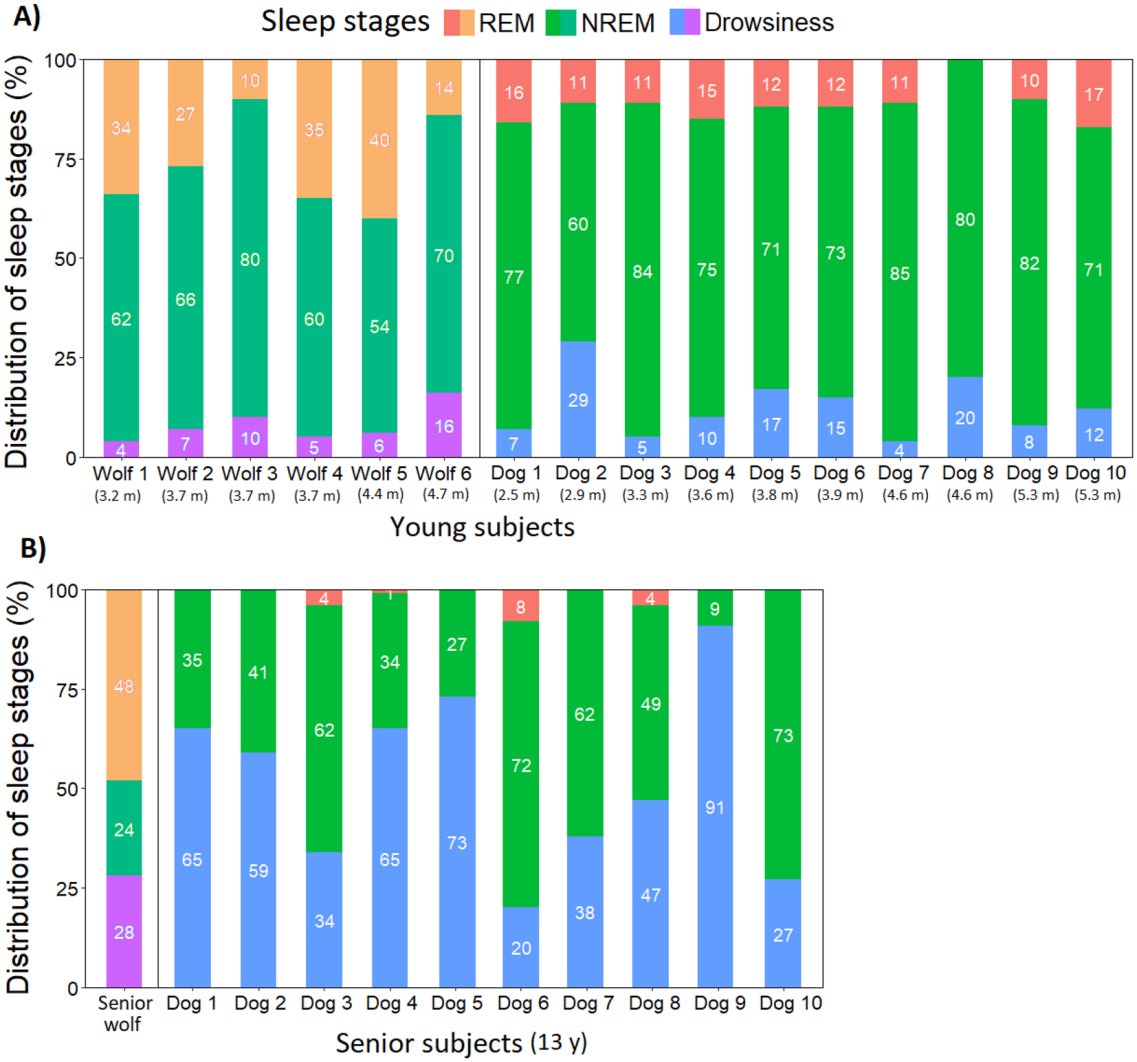
Distribution of sleep stages (REM, NREM, Drowsiness) in (**A**) young wolves and dogs and (**B**) Senior wolf and dogs. All data indicate the first hour (from the first drowsiness/NREM epoch) of sleep occasion 1.

Based on visual inspection, young wolves seem to have spent most of the time in NREM (54–80% of sleep time) and the least time in drowsiness (4–16% of sleep time), while the time spent in REM seems to be more variable, ranging between 10 and 40% (Fig. 3A). Considering the Senior wolf, (Fig. 3B), it seems that he spent most of the sleep time in REM (48% of sleep time) while the amount of NREM and drowsiness were similar (24 and 28%).

Data of young wolves and dogs seem to be similar regarding the proportion of time spent in drowsiness, although based on visual examination young dogs appear to have spent less time in REM and more time in NREM, compared to young wolves (Fig. 3A).

Considering the sleep of the seniors, dogs seem to have spent more time in drowsiness (20–91% of sleep time) and NREM (34–73% of sleep time) than the Senior wolf (Fig. 3B). However, they have barely entered REM sleep at all (2 senior dogs spent 4% and one senior dog spent 1% and 8% of their sleep time in REM), while half of the Senior wolf ‘s sleep time consisted of REM sleep (Fig. 3B and for the hypnogram see Fig. S4).

We also visualized the first and second sleep occasions of three wolves (Supplementary Fig. S5). Based on visual inspection, it seems that the sleep macrostructure of Wolf 6 remained stable, unlike those of Wolf 4 and Senior wolf. Wolf 4 spent less time in REM and more time in NREM and drowsiness on the second occasion, compared to the first one. While Senior wolf also spent less time in REM and more time in NREM on the second occasion, the time spent in drowsiness did not change between the two occasions (Supplementary Fig. S5).

### Power spectrum

Data of power spectrum variables indicate that both young wolves and dogs show a high proportion of delta power activity (Table 2). In senior subjects it seems that the proportion of delta power activity is lower (lowest in the first sleep measurement of the Senior wolf), while the proportion of the theta, alpha, sigma and beta frequency bands are higher compared to the young subjects. The individual relative power spectrum of delta (1–4 Hz), theta (4–8 Hz), alpha (8–12 Hz), sigma (12–16 Hz) and beta (16–30 Hz) ranges are visualized in Supplementary Fig. S6 (young animals) and in Supplementary Fig. S7 (seniors). In the case of young animals, the frequency range of 16–30 Hz was not visualized as it contained less than 0.03% of the whole relative power spectra.

**Figure 4:**
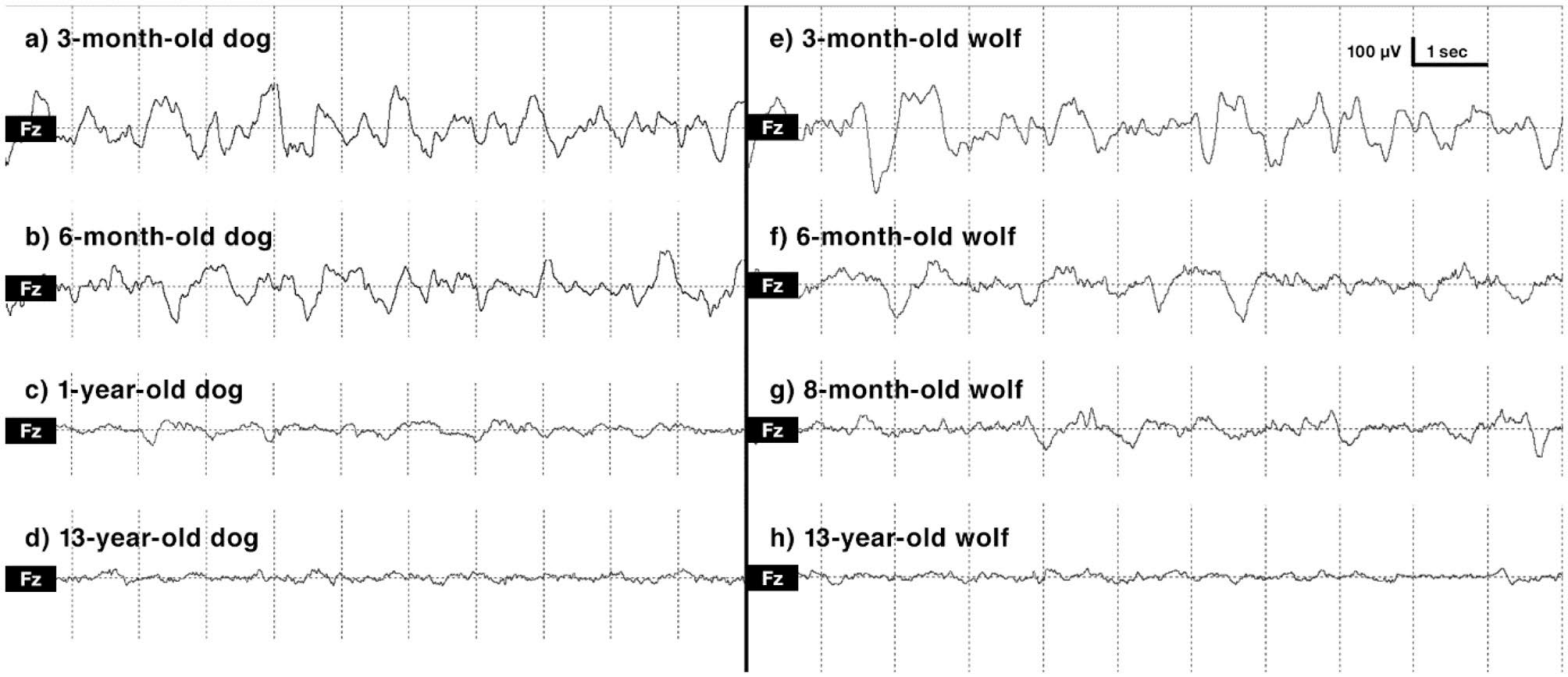
Representative EEG traces of NREM sleep from different ages of (a–d) dogs and (e–h) wolves. EEG traces of 6-month-old and 1-year-old dogs are from a previous dog polysomnography study^51^.

To further visualize differences in power spectrum variables, we included EEG traces of NREM sleep. Visual inspection of the sleep EEG indicates age-related differences. More specifically, prominent slow waves are observable in the EEG traces of young wolves and dogs, while these are absent in the EEG traces of aged subjects (Fig. 4).

## Discussion

To the best of our knowledge, our study is the first to explore the EEG of natural sleep in wolves using a fully non-invasive methodology. By registering both spectral and macrostructural sleep data, we provided a detailed description of the sleep EEG of six young and one senior wolf, also demonstrating the comparability of our procedure with other non-invasive sleep studies.

Analyzing the sleep of various different species is an invaluable source of information for a number of reasons. Besides extending our knowledge on the sleep of the species under investigation, it provides the opportunity for comparative studies, a tool helping us gain a broader understanding of the functions of sleep across animal groups and the ecological and evolutionary factors affecting them^10^. However, methodological differences between sleep experiments can greatly affect the data acquired and conclusions drawn from individual studies, decreasing their comparability^10^. The ‘gold-standard’ of determining and characterizing sleep states and architecture has been based on the neurological correlates of sleep, since behavioural observations alone may lead to misleading conclusions^10,59^. Traditionally, EEG in animals have mostly been recorded invasively, involving the surgical implantation of electrodes on the animals’ brain along with the restriction of free movement (e.g.^60–62^). Although these methods usually provide higher signal quality, they entail several costs including the need for surgical implantation of electrodes requiring special surgical and anesthetic equipment, basically restricting feasible subjects to a limited number of laboratory animals^63^. Additionally, as the general aim to apply more humane animal experimentation methods is progressively adopted worldwide, invasive methodologies have become more and more questionable from ethical and animal welfare point of view^12^, calling for the need of more widely applicable and reliable non-invasive procedures. A further important consideration is that it is increasingly recognized that non-invasive methodologies may be exceedingly important in translational research, more successfully connecting results from different animal species to human research^64^. Our non-invasive methodology offers a solution to many of the above-mentioned issues. As being fully non-invasive and without requiring any prior training, it can be applied under non-laboratory circumstances and to a wider range of animal species. As a result of these characteristics, it offers a more easily and flexibly applicable method than minimally invasive techniques used in a range of different species including barn owls^59^, wild sloths^65,66^, hibernating lemurs^67^ or dogs^68,69^. Additionally, since the method has been directly derived from the recording technique used in humans, it allows for a more direct comparison with human studies and application in translational research^16,55^.

The possibility to infer valuable information from the collected data is a further major aspect of any EEG experiment. Our methodology has proved to be a reliable source of EEG data and a basis for meaningful comparisons with other species as demonstrated by a line of different experiments conducted in dogs. For example, it has been shown in dogs that their sleep spectral feautres are predictive of their memory performances^70^ and that sleep spindles are associated with better learning^20,71^, similarly to humans^72,73^ and rats^74,75^. Different types of learning tasks have also been successfully carried out with wolves in comparative studies (e.g.^76,77^), thus combined with the currently described sleep EEG method it opens up the way to research on wolf memory consolidation. Another study showed that similarly to humans^78^, emotional pretreatment has an effect on the sleep macrostructure in dogs^19^. Interestingly, similarly to humans, so-called ‘first-night effect’-like sleep macrostructure changes have also been shown in dogs between repeated afternoon sleeps at the same unfamiliar location^28^, with certain differences between the two species^21^. Nevertheless, ‘first-night effect’-like changes have not been found in dairy cows^79^, and the limited wolf data in the current study also presents mixed findings regarding the potential difference between the first and second sleep occasions.

Although the sample size in the current study is too low and the age distribution of the subjects is highly skewed to draw comparative conclusions from our results, some conjectures may still be warranted from the trends shown in our data, at least in the studied age groups. We have found slight differences in the measured parameters, both between species and age groups. While young dogs and wolves show a similar distribution of sleep stages, the time spent in REM is less in dogs than in wolves, and this difference is even more conspicuous in the senior animals. This finding is especially intriguing since the amount of REM sleep has been linked to various different effects (and species) including neurodevelopment^80^, stress^81^, domestication^82^, but also memory consolidation or relative brain mass^83^. Interestingly, similar age-related slow wave sleep changes have also been found in humans, but several studies suggest that this reduction is more pronounced in men than in women^84,85^. We have also found age-related differences in the sleep EEG spectrum in both species, with the proportion of delta power, ‘slow wave’ activity being lower in senior animals. Although the literature is rather scarce regarding similar changes in non-human animals except for some notable examples^86^, a recent study has identified age-related sleep pattern changes in young dogs^51^. Specifically, between the ages of 8–14 months, larger-sized and older dogs had less delta and had more theta and alpha power activity. Older dogs exhibited greater sigma and beta power activity. Comparing the sleep macrostructure of different sleep occasions of the same individuals shows that there is high individual variability in the consistency of sleep architectures. This points to the important notion that in order to conduct comprehensive sleep studies, it may not only be important to collect data from more subjects, it may also be highly informative to repeat measurements on the same subjects.

The highly biased age distribution is an important limitation of our study, but at the same time, it also points out an important research-methodological conclusion. Unfortunately, collecting data on captive wolves is inherently difficult in this regard, since while young, socialized animals are more easily handled, the handling of adult animals may be highly variable depending on the individual. Thus, we suggest that using a reliable, easily applicable methodology across different studies—such as the methodology used in our study—allows for the valid combination of results from different studies and study-sites and to draw stronger scientific conclusions from those results. Such collaborative multi-site studies are gaining increasing popularity in cognitive and psychological research areas in an attempt to reach statistically meaningful sample sizes or to increase the generalizability of the results^87,88^. Larger sample sizes would also allow to control for important factors affecting sleep characteristics such as sex^89^ and age^86^. In the future, sleep measurements could also be complemented by the acquisition of further biosignals such as heart or breathing rate, extending our knowledge on the physiological changes associated with different sleep stages. Another limitation of our study is that the wolves we studied live in captivity, and we know that this may affect certain aspects of their sleep (e.g. sleep duration and quality^10,59,90^). However, the neurobiology of wild animals is notoriously difficult to study and investigating them in captivity may offer an acceptable compromise. In our specific case, collecting data from wolves offers the opportunity to compare the results with that of its domesticated relative, the dog. Since our wolves were socialized similarly to family dogs, this may help to complement our knowledge on the widespread effects domestication has on species, affecting a wide range of phenotypic features from behaviour to appearance^91^.

In the future, however, it would be imperative to further improve methodologies to be able to collect data from freely moving wild animals non-invasively. Research in this field has also seen major developments in recent years. For example, remote sensing techniques have advanced considerably^92,93^, EEG amplifiers have improved tremendously in terms of signal quality and size reduction (e.g.^94^) and new, more widely and easily applicable EEG electrodes have also been developed (e.g. dry, non-metallic, self-adhesive electrodes^95^).

In summary, our study not only highlights the possibility to gain a better understanding of the neurobiology of sleep in wolves or the changes associated with domestication, but studying the sleep of a so far unexplored species may also advance our knowledge in the neurobiology and functions of sleep in general. By promoting the use of a non-invasive EEG technique of high ecological validity and adequate signal quality, we aim to increase the welfare of participating animals, and set the methodological grounds for large-scale multi-site studies in order to achieve conclusive sample sizes and increase the replicability of studies.

## Supplementary Information

Supplementary Information.
