# Supplementary material for: Non-invasive sleep EEG measurement in hand raised wolves
Source: Sci Rep. 2022 Jun 13;12:9792. doi: 10.1038/s41598-022-13643-x (PMC9191399; doi:10.1038/s41598-022-13643-x)
Supplement: Supplementary file 1 — Supplementary Information. [file 41598_2022_13643_MOESM1_ESM.docx]

# SUPPLEMENTARY INFORMATION to:

Non-invasive sleep EEG measurement in hand raised wolves

Vivien Reicher^1,2^, Anna Bálint^2^, Dóra Újváry^3^, Márta Gácsi^2,3^

^1^ Doctoral School of Biology, Institute of Biology, Eötvös Loránd University, Department of Ethology, Budapest, Hungary

^2^ MTA-ELTE Comparative Ethology Research Group, Budapest, Hungary

^3^ Institute of Biology, Eötvös Loránd University, Department of Ethology, Budapest, Hungary

Author Notes

Conflict of interest:

The Authors declare no conflict of interest.

Correspondence to:

Vivien Reicher

Eötvös Loránd University, Institute of Biology, Department of Ethology

Pázmány Péter sétány 1/C

1117 Budapest, Hungary

Email: [vivien.reicher@ttk.elte.hu](mailto:vivien.reicher@ttk.elte.hu)

Phone: +36-1-411-6500 /Ext. 8788

Table S1

*Demographic data of wolves and dogs. “m” indicates months, “y” indicates years.*

| **Subject** | **Age** | **Sex** | **Reproductive status** | **Breed** | **Location and date of sleep EEG** |
| --- | --- | --- | --- | --- | --- |
| Wolf 1 | 3.2 m | female | intact | - | Wolfpark |
| Wolf 2 | 3.7 m | male | intact | - | Wolfpark |
| Wolf 3 | 3.7 m | male | intact | - | Wolfpark |
| Wolf 4 | 3.7 m | female | intact | - | Wolfpark |
| Wolf 5 | 4.4 m | female | intact | - | Wolfpark |
| Wolf 6 | 4.7 m | female | intact | - | wolfpark |
| Dog 1 | 2.4 m | female | intact | cocker spaniel | University |
| Dog 2 | 2.9 m | male | intact | labrador retriever | University |
| Dog 3 | 3.3 m | female | intact | border terrier | University |
| Dog 4 | 3.6 m | male | intact | border collie | University |
| Dog 5 | 3.8 m | male | intact | tervueren | University |
| Dog 6 | 3.9 m | female | intact | rough collie | University |
| Dog 7 | 4.6 m | female | intact | golden retriever | University |
| Dog 8 | 4.6 m | male | intact | boxer | University |
| Dog 9 | 5.3 m | male | intact | australian shepherd | University |
| Dog 10 | 5.3 m | female | intact | golden retriever | University |
| Senior wolf | 13 y | male | neutered | - | University |
| Senior Dog 1 | 13 y | female | neutered | mix | University |
| Senior Dog 2 | 13 y | female | neutered | border collie | University |
| Senior Dog 3 | 13 y | female | neutered | beagle | University |
| Senior Dog 4 | 13 y | female | neutered | border collie | University |
| Senior Dog 5 | 13 y | female | neutered | mix | University |
| Senior Dog 6 | 13 y | female | neutered | dalmatian | University |
| Senior Dog 7 | 13 y | female | neutered | mix | University |
| Senior Dog 8 | 13 y | male | neutered | mix | University |
| Senior Dog 9 | 13 y | male | neutered | mix | University |
| Senior Dog 10 | 13 y | male | neutered | border collie | University |

EEG recording:

The two electrodes placed on the right and left zygomatic arch next to the eyes (F8, F7) and the scalp electrodes over the anteroposterior midline of the skull (Fz, Cz) were referred to the G2 reference electrode in the posterior midline of the skull (occiput; external occipital protuberance). The ground electrode (G1) was attached to the left *musculus temporalis*. In all dogs and wolves the frontal electrode (Fz) was active, thus data from this electrode were used for spectral analyses. We used gold-coated Ag/AgCl electrodes, secured by Signa Spray Electrode Solution (Parker, United States) and EC2 Grass Electrode Cream (Grass Technologies, United States). Impedance values were kept under 20 kΩ during the recordings.

Recordings were obtained with one of the following two technical arrangements (as one EEG equipment was mobile, while the other was not):

(1) In the case of 9 dogs the signal was collected, amplified and digitised at a sampling rate of 1000 Hz/channel, using the 40-channels NuAmps amplifier (© 2018 Compumedics Neuroscan). The signal was DC-recorded, saved in .cnt format using the Scan 4.3 Acquire software (© 2018 Compumedics Neuroscan) and converted to .edf format using the MatLab EEG Toolbox.

(2) In the case of all wolves and 11 dogs the signals were collected, pre-filtered, amplified and digitized at a sampling rate of 1024 Hz/channel, using the 25 channel SAM 25R EEG System (Micromed, Mogliano Veneto, Italy), and the System Plus Evolution software with second-order filters at 0.016 Hz (high pass) and 70 Hz (low pass). Data was exported in .edf format.

To correct for differences in EEG filter characteristics across recording devices, a calibration process was implemented. Specifically, a waveform generator at the Fz electrode input of both devices was used to apply 40 and 355 μV amplitude sinusoid signals at various amplitudes (0.05 Hz, every 0.1 Hz between 0.1–2 Hz, every 1 Hz between 2–20 Hz, every 10 Hz between 10 Hz-100 Hz). The amplitude reduction rate for each recording system was determined by calculating the proportion of digital (measured) and analog (generated) amplitudes of sinusoid signals. Next, amplitude reduction rates were calculated for each device and EEG spectrum amplitudes were corrected by dividing such calculated values by the obtained amplitude reduction rate for the recording system.


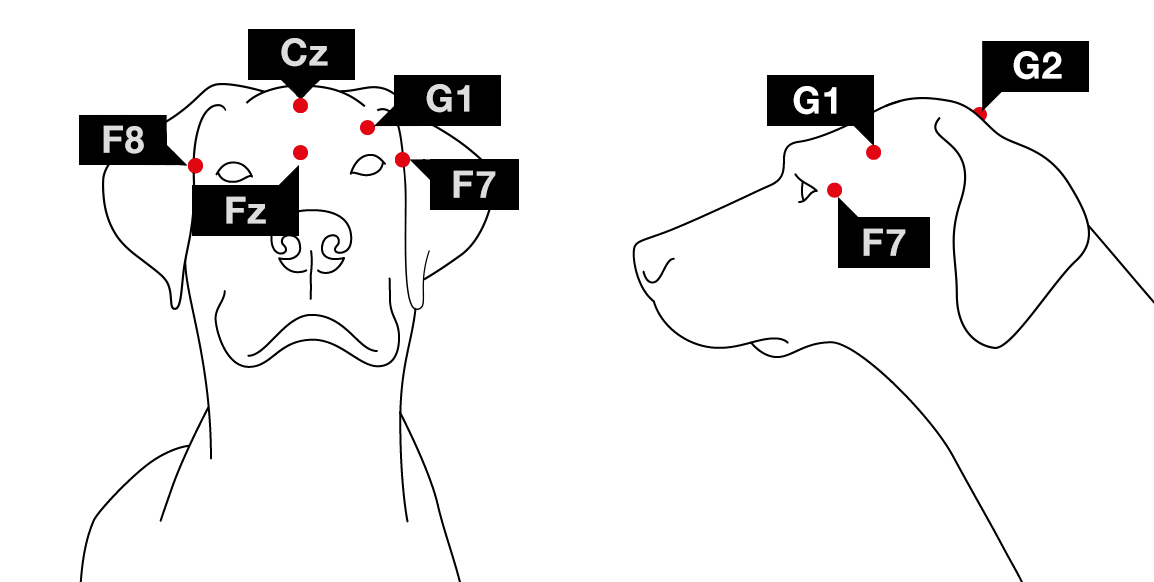


Figure S1: Placement of the electrodes (Fz-Cz: frontal and central midline; F7-F8: left and right electrodes placed on the zygomatic arch; G2: reference electrode; G1: ground electrode)


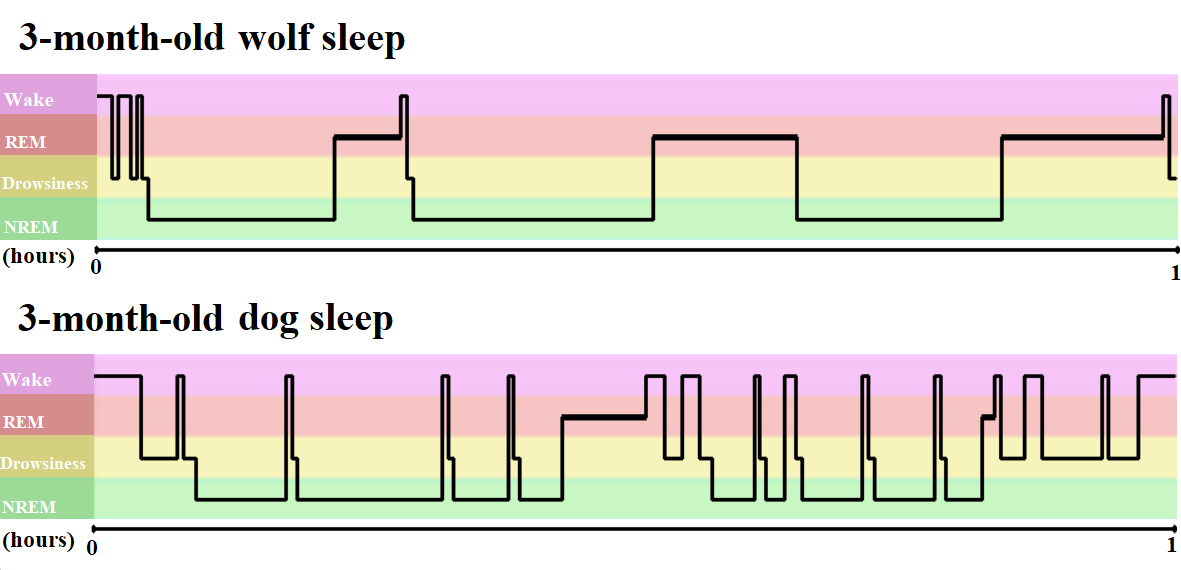


Figure S2: Example hypnograms of typical one-hour-long sleep recordings of a wolf and a dog


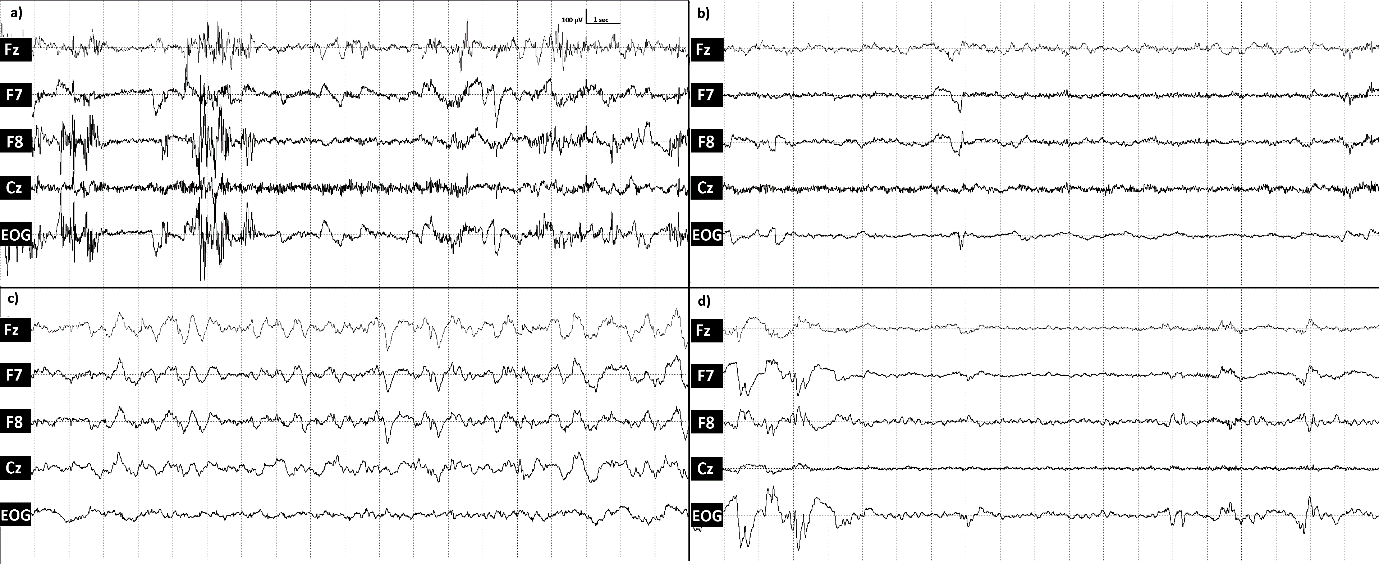


Figure S3: Representative EEG traces of different sleep stages of a young wolf; a) wake, b) drowsiness, c) NREM and d) REM sleep.


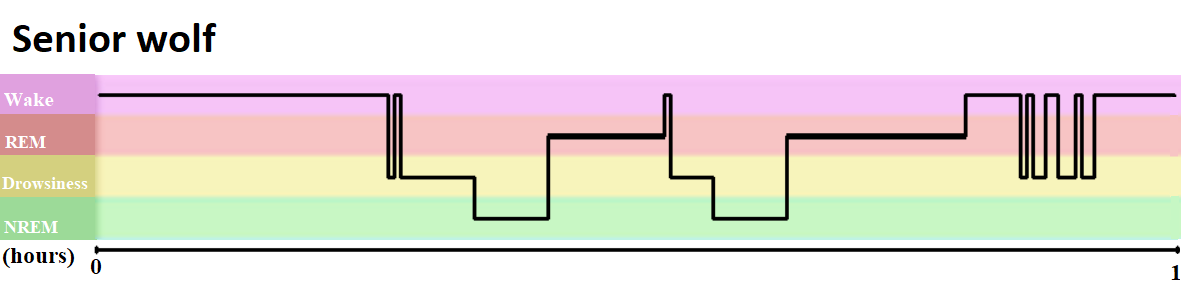


Figure S4: hypnogram of the senior wolf’s first sleep recording.


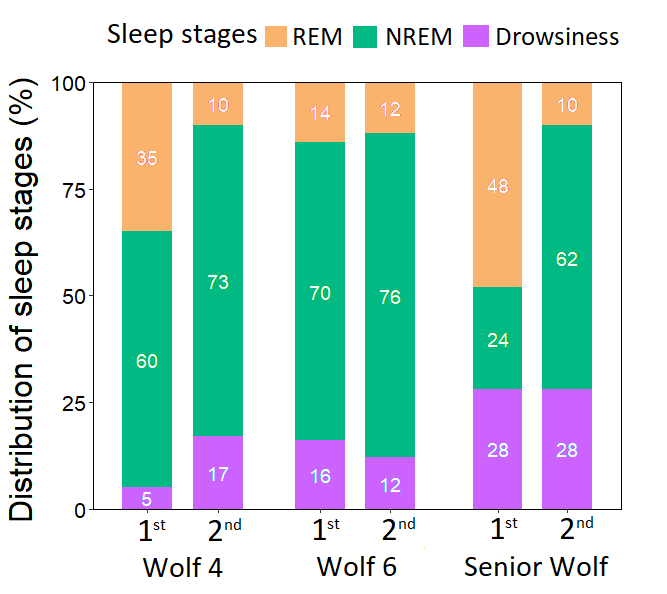


Figure S5: Distribution of sleep stages (REM, NREM, Drowsiness) in the first and second sleep occasions. All data indicates the first hour (from the first drowsiness/NREM epoch) of sleep.


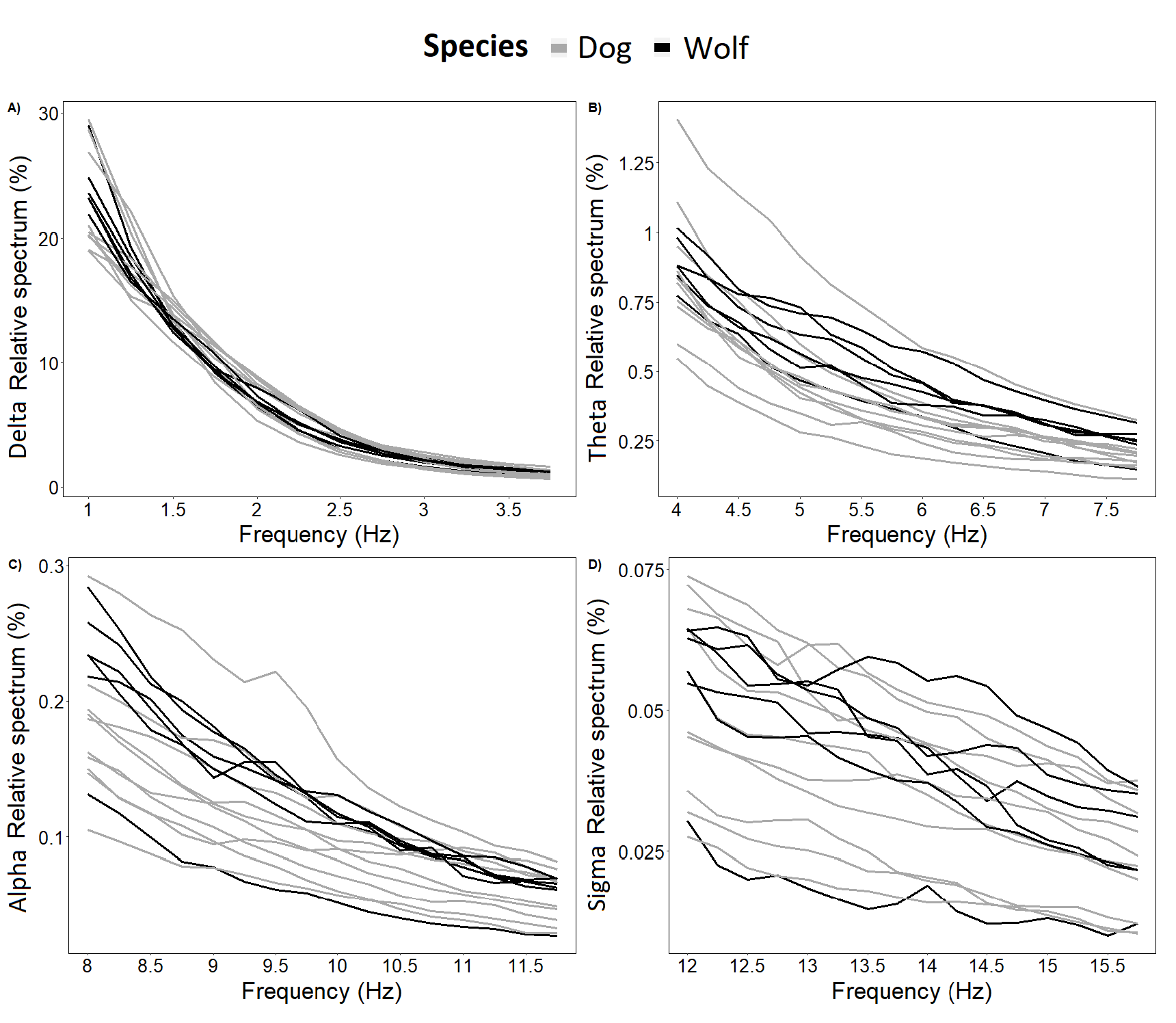


Figure S6: Relative power spectra of NREM sleep in young dogs and wolves in the frequency ranges of A) delta, B) theta, C) alpha, D) sigma.


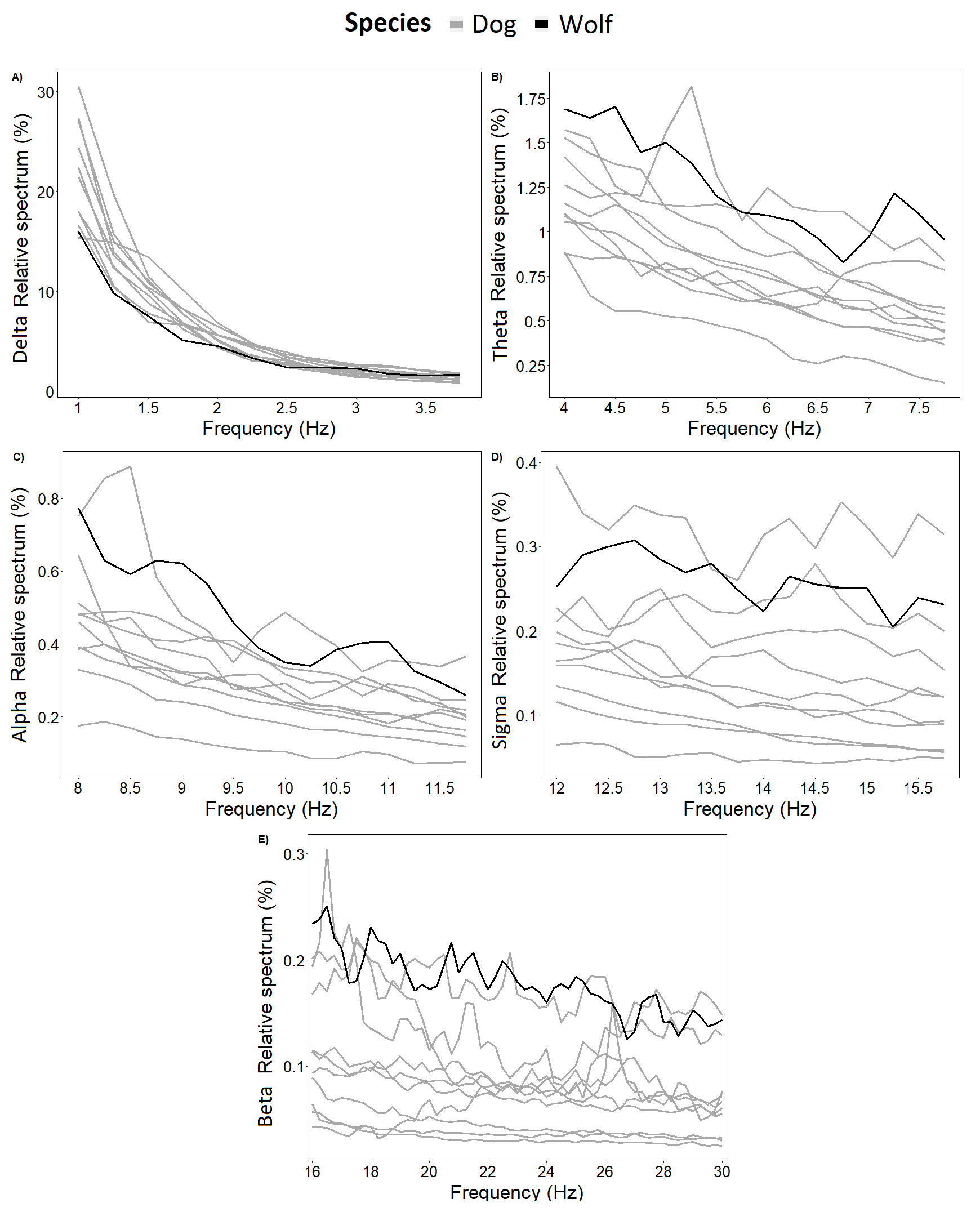


Figure S7: Relative power spectra of NREM sleep in senior dogs and one senior wolf in the frequency ranges of A) delta, B) theta, C) alpha, D) sigma and E) beta.
